# Diffusion Control of Organic Cathode Materials in Lithium Metal Battery

**DOI:** 10.1038/s41598-019-38728-y

**Published:** 2019-02-04

**Authors:** Rachel L. Belanger, Basile Commarieu, Andrea Paolella, Jean-Christophe Daigle, Stéphanie Bessette, Ashok Vijh, Jerome P. Claverie, Karim Zaghib

**Affiliations:** 1 Université de Sherbrooke, 2500 Blvd de l’Université, Sherbrooke, Québec, J1K 2R1 Canada; 2 Center of Excellence in Transportation Electrification and Energy Storage (CETEES), Hydro-Québec, 1800, Lionel-Boulet Blvd., Varennes, Quebec, J3X 1S1 Canada

## Abstract

Organic cathode materials for lithium batteries are becoming increasingly popular because they have high theoretical redox voltage, high gravimetric capacity, low cost, easy processing and sustainability. However, their development is limited by their solubility in the electrolyte, which leads to rapid deterioration of the battery upon cycling. We developed a Janus membrane, which consists of two layers – a commercial polypropylene separator (Celgard) and a 300–600 nm layer of exfoliated graphite that was applied by a simple and environmentally friendly process. The submicron graphite layer is only permeable to Li^+^ and it drastically improves the battery performance, as measured by capacity retention and high coulombic efficiency, even at 2C rates. Post-mortem analysis of the battery indicates that the new membrane protects the anode against corrosion, and cathode dissolution is reduced. This graphite-based membrane is expected to greatly expedite the deployment of batteries with organic cathodes.

## Introduction

Energy storage technology is a critical research area for the success of portable electronic devices^1,2^ and electrical transportation^3^. Such applications need affordable, durable, safe and environmentally friendly battery materials^4^ with high energy density^5^. Organic cathode materials are currently promising candidates because they fulfill most of these requirements for an active battery material^6^. In comparison to inorganic-based cathode materials (such as LiCoO_2_ or LiFePO_4_), organic cathode materials represent a sustainable alternative that does not require energy-intensive transformations^7^. Furthermore, using organic materials with low molecular weight enhanced the battery energy because the mass of active material per exchanged electron is reduced^8–10^. Organic cathode materials can also reach high redox potentials, most notably when decorated by electronegative functional groups^11^. However, they usually suffer from a major limitation, namely their solubility in organic electrolytes^12^. Even a very low solubility translates into a decreased capacity upon cycling due to the loss of active material.

Among organic cathode materials, conjugated carbonyl compounds have been intensively scrutinized because of a combination of desirable properties, such as low cost, good theoretical capacity, reversible oxidative behavior, high discharge potential and commercial availability^13–16^. For example, 3, 4, 9, 10-perylene-tetracarboxylic-dianhydride (PTCDA) is an inexpensive red pigment that is widely investigated as an active material for energy devices (solar cells^17^, battery^18,19^). However, Li-PTCDA batteries suffers from poor and irreversible cycling stability due to the dissolution in the electrolyte^20^. Even more problematic, the dissolved PTCDA migrates through the porous separator and deposits on the anode surface causing irreversible damage^21^. In order to solve this problem, chemical modifications such as polymerisation^22,23^, functionalization^24,25^ and immobilization on carbon materials^26,27^ have improved cycling stability and coulombic efficiency. However, these modified cathode materials, which are often prepared by complex processes, contain considerable amounts of inactive mass that cause a decreases of the energy density.

Carbon-based membranes are known to suppress polysulfide shuttling behavior leading to enhance the electrochemical performance of lithium-sulfur (Li-S) batteries^28–32^. Here, we show that carbon materials can also be used as a selective interlayer for Li^+^ ions in a Li-PTCDA battery to enhance cycle life. For this purpose, we developed a low cost and solvent-free method by applying a thin graphite layer on one side of a commercially available polypropylene (PP) microporous membrane (Celgard 3501, referred here as Celgard). The modified separator, coined as G-separator, acts as a selective layer for the transport of Li^+^ between electrodes, and protects the lithium anode from corrosion by inhibiting the diffusion of dissolved PTCDA. This graphite interlayer, which adds less than 0.5% to the weight of the battery material, significantly improves cycling stability with a coulombic efficiency near 100% after 100 cycles. The fabrication of the G-separator does not require any solvent or binder, chemical modification or any energy-consuming curing process. Thus, we envision that the G-separator can be implemented on a large scale, leading to the deployment of lighter, more sustainable batteries.

## Results and Discussions

**Figure 1:**
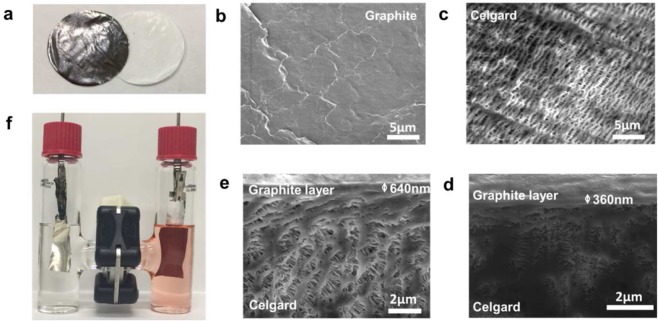
(**a**) Image of the Celgard and the G-separator. SEM images of (**b**) graphite side of the G-separator, (**c**) a Celgard and the (**d**) cross-section of G-separators with 360 nm (G-separator A) and (**e**) 640 nm (G-separator B) thick graphite layers. (**f**) Li-PCTDA battery with G-separator mounted in a H-cell configuration and cycled for 40 hours, showing the absence of diffusion of PCTDA through the G-separator.

During the smearing step in the G-separator preparation, the physical friction results in the formation of a continuous and non-porous film of graphite (Fig. 1a) that does not affect the original flexibility of Celgard (Supplementary Fig. 1). Scanning electron microscopy (SEM) of a G-separator shows that the graphite interlayer (Fig. 1b) is smooth, uniform in thickness, devoid of cracks or aggregates and it completely covers the porous morphology of the Celgard (Fig. 1c).The thickness of the graphite layer is dependant of the smearing time (see supporting information). The dense layers of graphite have an average thicknesses of 360 ± 50 nm (Fig. 1d) and 640 ± 70 nm (Fig. 1e), which correspond to an additional mass of 2% and 4% for the membrane (0.25 and 0.5%, respectively, relative to the battery mass). The scotch tape test qualitatively confirmed that graphite has good physical adhesion to the substrate. Raman spectroscopy showed that the coating process resulted in a higher proportion of edge defects, as indicated by a higher ratio I_D_/I_G_ (Supplementary Fig. 2)^33^. Such behavior is expected because the mechanical drawing process causes a misalignment of the graphite leaflets^34^. The affinity of the G-separator with the electrolyte was evaluated by contact angle analysis (Supplementary Fig. 3). The graphite on the G-separator exhibits a contact angle of 21.5°, which is less than the angle measured with the Celgard (35°) and indicates good wetting properties of graphite interlayer.

The use of conductive carbon on a porous membrane can cause internal problems related to the electrical isolation of the separator. The presence of graphite in the pores of the separator can electronically connect the two electrodes and cause short circuits. The interfacial stability can be evaluating using galvanostatic cycling and electrochemical impedance spectroscopy (EIS) of one Li|G-Separator|Li symmetrical cell. The cell was cycling at 0, 5 mA cm^−2^ for 867 h to evaluate the voltage fluctuation that can be cause by internal short circuits (Supplementary Fig. 4a). The long cycling stability demonstrates a high stability of the graphite layer. To support these results, EIS was conduct before cycling and after 100 h and 867 h (Supplementary Fig. 4b). These resistance profiles can support that there is no electrical bridge between the two lithium electrodes and that the cell still operate even after 867 hours.

The permeability of the G-separator to PTCDA was evaluated using an H-cell configuration. As shown in Fig. 1f, the G-separator is not permeable to PCTDA while Celgard is permeable (see Supplementary Fig. 5). Indeed, cycling an H-cell for 40 hours between 1.6–3.2 V induces the dissolution of PCTDA in the cathode compartment, as shown by its characteristic absorption at λ = 450–600 nm^35^. With Celgard, this characteristic absorption is observed in both compartments, due to diffusion of PTCDA through the porous separator. On the other hand, with the G-separator, the anode compartment remains free of chromophore, indicating that the graphite layer blocks the diffusion of soluble PTCDA.

**Figure 2:**
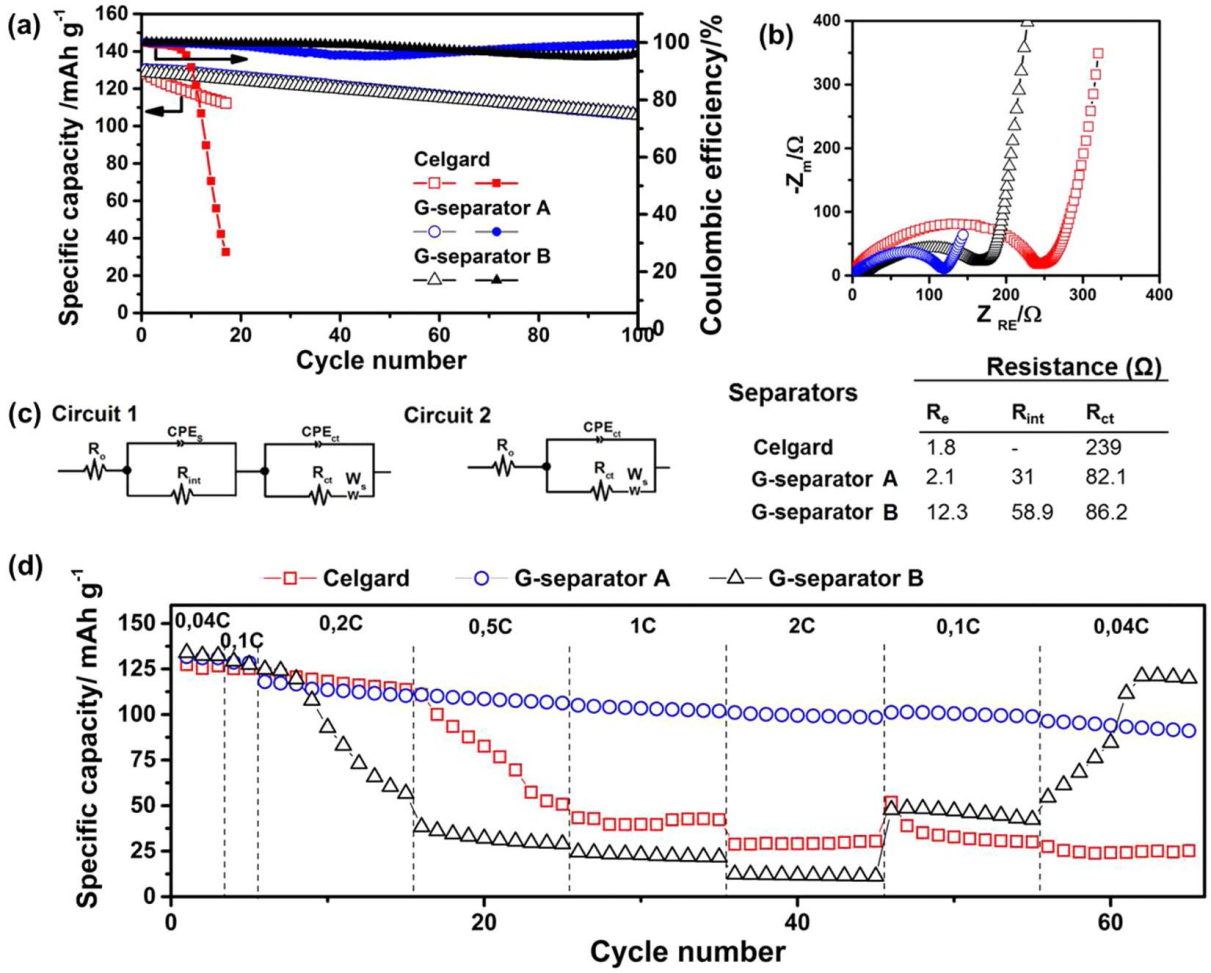
Electrochemical analysis of Li-PTCDA batteries using Celgard, G-separator A and G-separator B. (**a**) Cycling performance at 0.10C. (**b**) Impedance spectra of fresh cells, (**c**) their equivalents circuits and resistance values. (**d**) The rate capabilities of the cells.

The cycling stability of Li-PTCDA batteries with Celgard and G-separators (340 and 640 nm layers) was measured at 0.1C (1C = 0, 136 mA g^−1^) (Fig. 2a). The initial capacities are similar 128.5 +/− 1.5 mAh g^−1^, and the thickness of the graphite layer does not impact the capacities at this current density. With G-separators, a capacity of 106 mAh g^−1^ (80% retention) and a Coulombic efficiency of 97% is retained after 100 cycles. By contrast, with Celgard, the battery suffers from irreversible capacity loss and the Coulombic efficiency plummets to 27% after 17 cycles. The cycle life of the cell using a G-separator was also evaluated at the current rate of 1C (Supplementary Fig. 6). More than 300 cycles was achieved with a small capacity loss of 18% and high efficiency near 99%. This confirms the stability and the conservation of the barrier property of the graphite layer. Thus, the presence of a thin graphite interlayer on Celgard definitively improves the cyclability of the Li-PTCDA battery.

The effect of the graphite layer on the electrochemical kinetics was assessed by electrochemical impedance spectroscopy (EIS). The Nyquist plots and the values of the different parameters obtained through simulation of an equivalent circuit are shown in Fig. 2b,c. The intercept with the Z’ axes at high frequencies, R_e_, corresponds to bulk resistance of the cell. This value is only marginally affected by the presence of the G-separator, most particularly for the 360 nm separator, which indicates that the G-separator does not add significantly to the ohmic loss in the three batteries. The R_int_ corresponds to the resistance of the interface layer of all electrodes, and R_ct_ is associated to the charge transfer resistance. The ionic conductivity of the Celgard and the G-separator is 2.8 10^−4^ S.cm^−1^ and 3.1 10^−4^ S.cm^−1^, respectively, indicating that the graphite layer does not significantly impedes Li^+^ transport under operating conditions. Furthermore, graphite is an electronic conductor with an electronic conductivity of 760 S.cm^−1^, as determined by four-point probe measurement. The R_ct_ for the batteries with G-separators (82.1 Ω and 86.2 Ω) is considerably lower than that for Celgard (239 Ω), which is attributed to several factors: the better electrolyte wettability of the G-separator and the anticipated affinity of the graphite layer toward the PTCDA cathode^36^. In addition, the electronic contact between graphite and the cathode conductive materials functions as an internal current collector^37^. Consequently, the total resistance (summation of R_e_, R_ct_ and R_s_) is lower by 47% and 65%, respectively, for the batteries with G-separators (A and B) compared to the battery with Celgard.

The rate capabilities of batteries with Celgard and both G-separator cells were evaluated by cycling at different C-rates (Fig. 2d). At low current densities, such as 0.04C and 0.1C, the Celgard and G-separators show similar capacities (130 ± 3 mAh g^−1^ corresponding to 97% of the theoretical capacity of 136 mAh g^−1^). However, when the C-rate increases to 2C, the capacity of the batteries with Celgard and the G-separator B drastically decreases. When a rate of 0.04C is applied again, the Celgard battery exhibits a capacity of 24 mAh g^−1^, indicating 81% loss of initial value. However, the battery with a 640 nm G-separator recovers to a capacity of 120 mAh g^−1^, which corresponds to 90% of its initial value. The performance of the battery with the Celgard separator rapidly degrades due to PCTDA diffusion from cathode to anode. With the 640 nm G-separator, the capacity loss at high current densities is likely due to the slow diffusion through the thicker graphite membrane. Using a thinner graphite layer (360 ± 50 nm), the battery retains its capacity even at 2C. The Li^+^ diffusion time is expected to scale with the square of the thickness of the graphite layer, thus explaining the large difference in capacities between the G-separators A and B. Overall, the high electronic conductivity of the graphite layer and the shorter diffusion length in the G-separator A ensure rapid kinetics, which is a key advantage for fast-charging batteries.

Why is the G-separator so successful in ensuring ionic transport while also preventing the diffusion of PCTDA to the anode? To understand the possible mechanism of lithium diffusion through graphite layer, solvent permeability of the G-separator was investigated. An H-cell with no electrodes and a solution of LiPF_6_ in carbonates was used, with one of the compartments containing 2 wt.% of vinylene carbonate (VC). The other compartment remains free of VC after 24 hours when a G-separator is used, whereas an equilibrium concentration is reached with Celgard (Supplementary Fig. 7). Unlike Celgard, the graphite layer is non-porous, thus impermeable to solvent. In another experiment, the H-cell contains a 1M solution of sodium bis(trifluoromethanesulfonyl)imide (NaTFSI) in one compartment, and 1M LiTFSI in the other. After 72 hours, less than 0.03% Li^+^ (corresponding to the limit of detection) diffused in the NaTFSI compartment, as shown by ^7^Li NMR (Supplementary Fig. 8). By contrast, Celgard alone is permeable to solvent and ionic species (Supplementary Figs 5 and 6).

**Figure 3:**
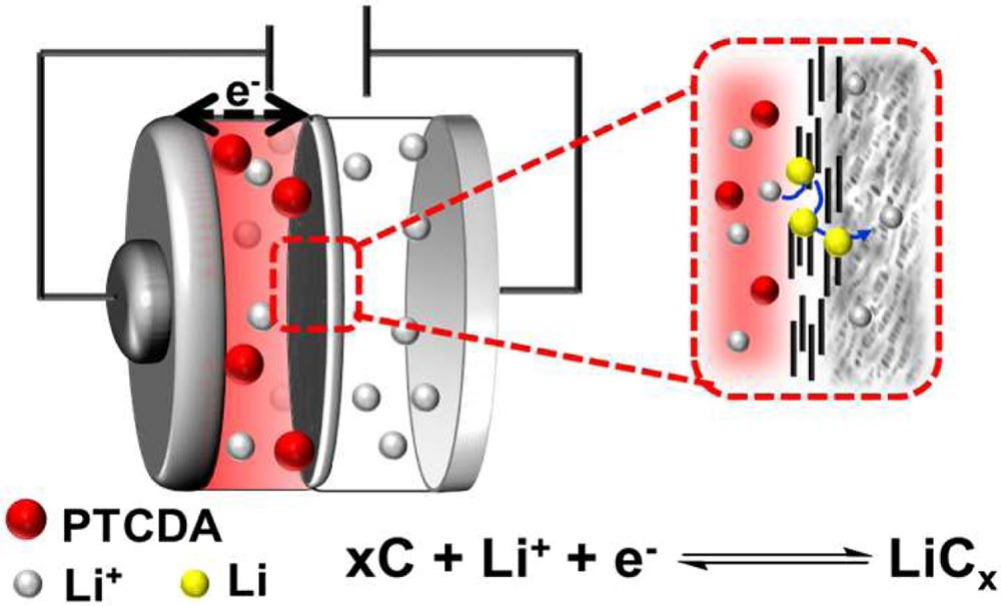
Schematisation of the Li-PTCDA battery containing a G-separator.

The graphite in the G-separator is an electronic conductor and this layer is in contacts with the stainless-steel container of the battery (cathode current collector). It is well known that lithium intercalates in graphite, leading to the formation of LiC_x_ (x: ∞, …, LiC_12_, LiC_6_)^38^. A battery containing the G-separator but no PTCDA cathode was cycled in the same potential range as the Li-PTCDA batteries presented above. This battery had a small initial capacity of 3.9 mAh g^−1^, which corresponds to 0.17% Li intercalated by graphitic carbon (Supplementary Fig. 7). Thus, the initial stage for good operation of the battery is the reductive intercalation of Li^+^ in the G-separator until percolation is reached, ie., until a continuous pathway through the graphite layer was created to allow lithium diffusion (Fig. 3). Based on the capacity measured on the battery containing a G-separator but no PCTDA cathode, this percolation threshold is reached when the graphite contains less than 1% intercalated lithium relative to carbon. Considering that the graphite layer represents less than 1% of the battery weight, the number of lithium intercalated in the graphite layer is very much smaller than the total number of lithium ions that migrate between the electrodes compartments. Overall, the small number of intercalated lithium in the non-porous graphite layer is sufficient to permit the rapid transport of Li^+^ between both electrodes. Remarkably, the transport of other species in the non-porous graphite layer such as PCTDA is prevented.

The post-mortem analysis on cycled Li-PTCDA batteries was performed using a combination of SEM coupled with Energy Dispersive Spectroscopy (EDS), Time-of-Flight Secondary Ion Mass Spectrometry (TOF-SIMS) and Fourier Transform Infrared (FT-IR) spectroscopy. The batteries with the Celgard and the G-separators were cycled for 65 cycles before disassembling (Supplementary Fig. 10). Visually, red material (PTCDA) is observed in the Celgard, whereas virtually no color is observed in the G-separator. Attenuated total reflectance FT-IR analysis showed that both surfaces of cycled Celgard separators contain PTCDA, as indicated by the presence of the characteristic 1750 cm^−1^ carbonyl band (see Supplementary Fig. 11)^39^. No PCTDA was detected on the anode side of the G-separator, indicating that the graphite layer blocked PTCDA diffusion under cycling conditions.

**Figure 4:**
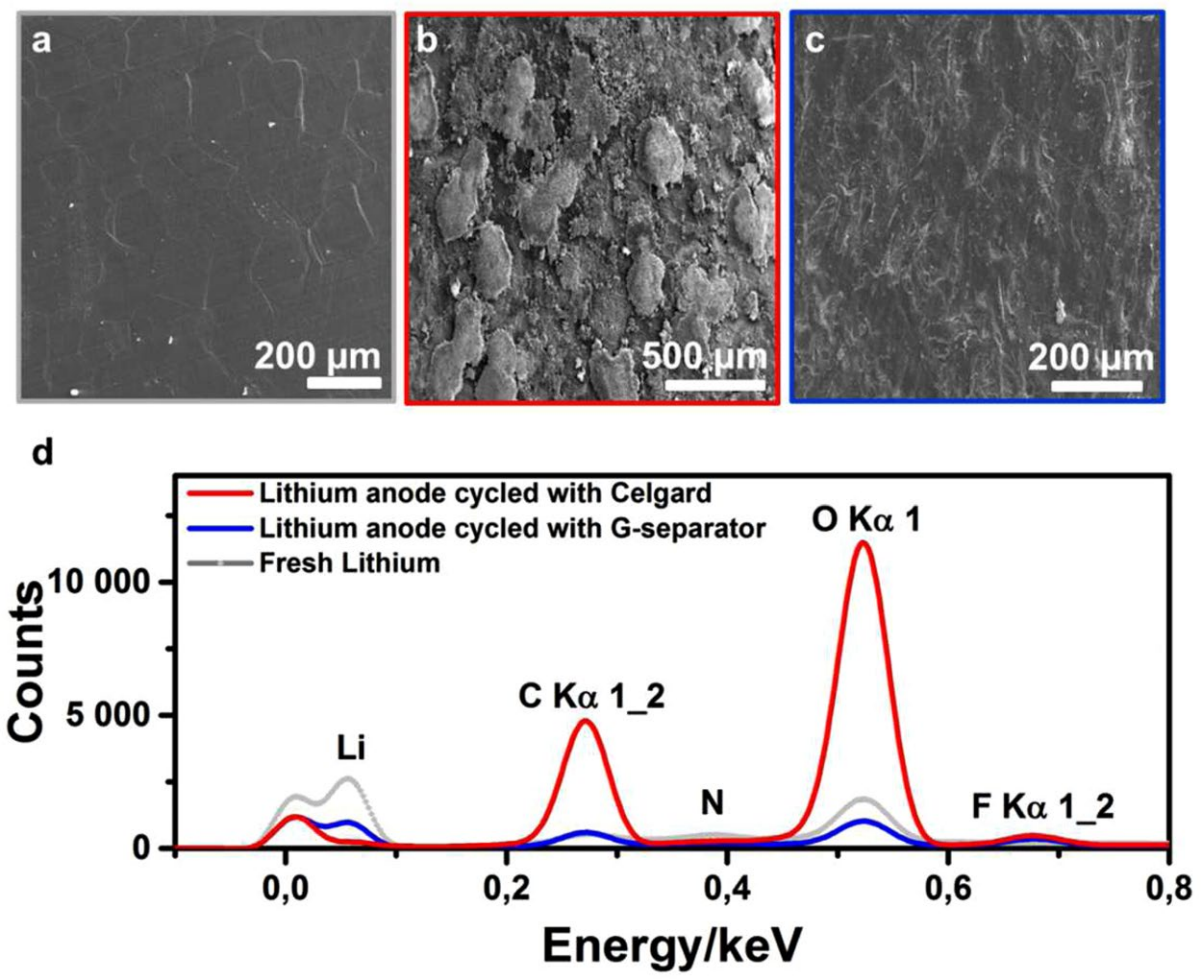
SEM images of (**a**) fresh lithium foil, cycled Li anode with (**b**) Celgard separator, (**c**) G-separator and (**d**) their corresponding EDS spectra.

Figure 4a–c display the SEM images of the surface of fresh lithium and lithium anodes cycled with Celgard and G-separator, respectively. A large amount of material was deposited on the Li surface that was cycled with Celgard separator, which is likely responsible to its poor cycle life^40^. Lithium metal batteries are known to suffer from dendrite formation due to a lithium striping-plating process^41,42^. SEM imaging at high magnification reveals dendrite-like needles on the anode in the Celgard-containing cell (Supplementary Fig. 12). By contrast, the anode in the cell containing G-separator is totally free of such dendritic structures. Energy-dispersive spectrometry analysis reveals the presence of lithium, carbon, oxygen and fluorine on the anodes in both cells (Fig. 4d). The anode cycled with the Celgard separator has high carbon and oxygen signals but a very low signal for lithium, whereas the opposite is observed for the anode cycled with the G-separator. These results corroborate the presence of large deposits observed at the surface of the anode cycled with Celgard. The EDS spectrum of the anode cycled with the G-Separator is similar to the fresh lithium anode, with a slight difference in intensity that is likely due to formation of a SEI^43^.

**Figure 5:**
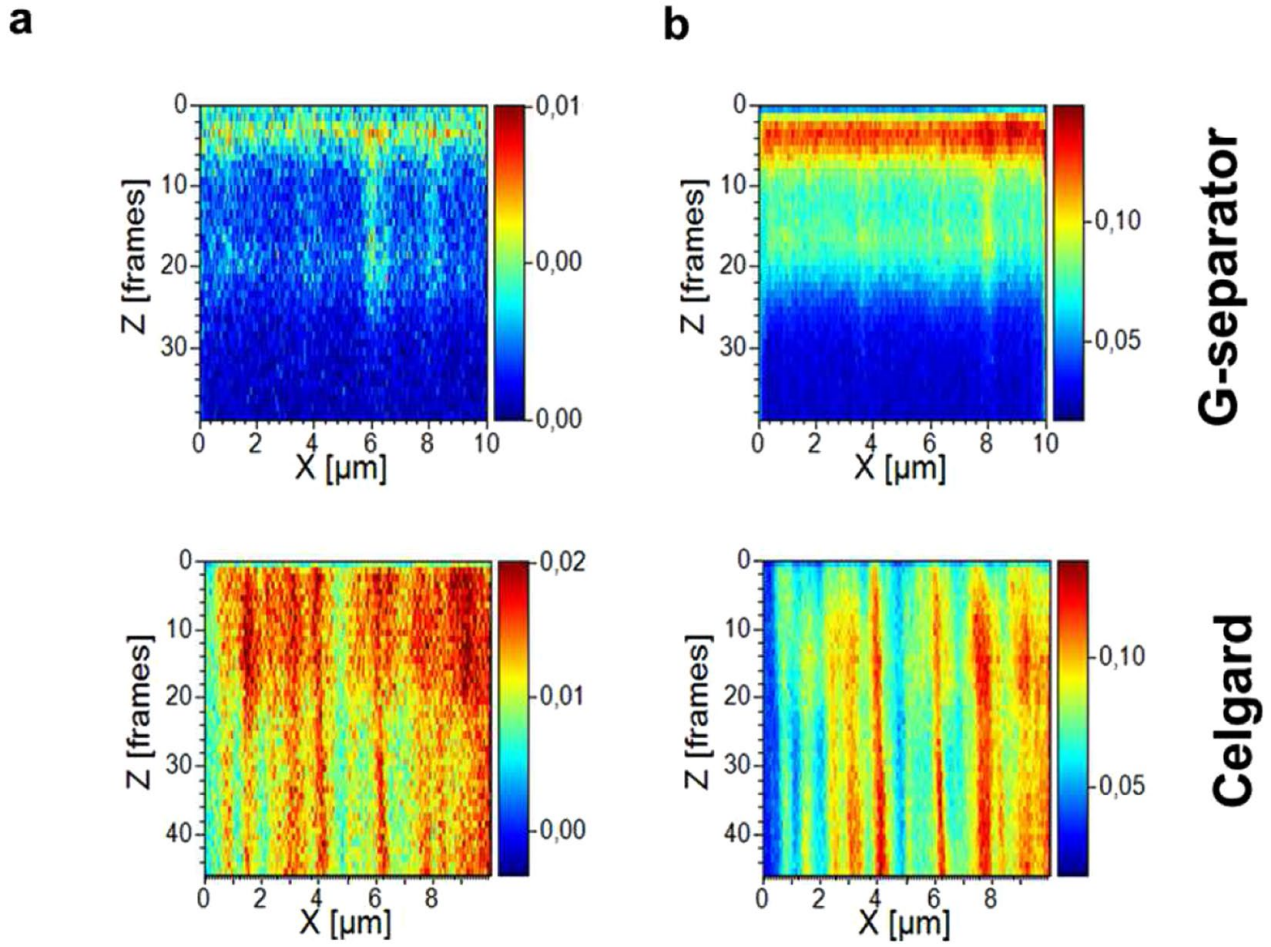
Depth profile of (**a**) m/z = 12, C^−^ and (**b**) m/z = 16, O^−^ of the anodes cycled with G-separator (top) and Celgard (bottom).

In order to probe the bulk composition of the anode after cycling, the anodes were analysed by TOF-SIMS (see Supplementary Table 1 for a list of major detected fragments). Figure 5 presents the z-distribution maps of C^−^ (m/z = 12) and O^−^ (m/z = 16) measured on anodes cycled with the Celgard and G-separator. In TOF-SIMS, the sputtering rate depends on various factors, so a precise relationship between the number of frames in the z-profile and depth is a priori not known, but it can be estimated using SEM images of the holes created by the bombardment of Ga^+^ ions (Supplementary Fig. 13). On the anode cycled with Celgard, 40 frames correspond to a depth of ca 1 µm, thus yielding an average 25 nm per frame. This anode shows that species containing carbon and oxygen have penetrated deeply into the surface to form channel-like structures that are approximately 1 µm wide and which fully span through the z-profile (that is to say they are at least 1 µm deep). By contrast, no such channels are observed in the anode cycled with the G-separator, but a SEI that is approximately 50 to 100 nm thick is observed on the anode surface. Thus, TOF-SIMS analysis indicates that the Li anode cycled with the G-separator remains intact, whereas considerable pitting corrosion is observed in the Li anode cycled with Celgard.

Lithium nitrate (LiNO_3_) is often used as an electrolyte additive in Li-S batteries to passivate the anode via formation of a thick lithium nitrous oxide (LiNO_x_) layer^44^. This thick SEI is known to prevent dissolved polysulfides from reacting with the lithium anode. In a Celgard Li-PCTDA battery, the addition of 1% LiNO_3_ improves the coulombic efficiency from 27% to 96% after 17 cycles at 0.1C (Supplementary Fig. 14). Thus, the lower coulombic efficiency observed with the Celgard separator in the absence of LiNO_3_ corresponds to the corrosion of the anode by PTCDA, as also evidenced in Figs 4 and 5. However, LiNO_3_ does not prevent the decrease of specific capacity which is directly related to the dissolution of PTCDA. By contrast, with the G-separator, the solubilisation of PTCDA is limited, as shown by 20% loss of capacity after 100 cycles (at 0.1C). Indeed, the presence of the graphite layer confines the soluble PTCDA to the cathodic compartment, thus reducing the volume of electrolyte available for dissolution. When this barrier is not present, large amounts of PTCDA can dissolve in the electrolyte, in the pores of the Celgard separator and precipitate either at the separator surface (as shown in the post-mortem analysis, Supplementary Figs 8 and 9), or at the anode surface (Figs 4 and 5).

## Conclusion

In summary, we developed a strategy to address the poor cycling performance of Li-PTCDA batteries. The aim of our study was to investigate the fundamental properties of graphite interlayer for organic cathode diffusion issues during cycling. A solvent-free graphite deposition process was used to form a Janus membrane consisting of a porous, flexible and mechanically stable polypropylene layer on one side and a thin, non-porous and continuous layer of graphite on the other side. This G-separator prevents PTCDA molecules from diffusing through the separator and reacting with the anode, thus producing batteries that have superior cycle performance, even at 2C rates although their energy density is still far from an industrial application. The separator modification that we propose is expected to open new simple routes to address the dissolution issues with many active materials used in batteries.

## Methods

### Celgard Modification

The G-separator was prepared by smearing a graphite powder, purchase from HITACHI, directly on one side of a typical commercial PP membrane (Celgard 3501) for 5 minutes to obtained a ~360 ± 50 nm and 10 minutes for a ~640 ± 70 nm graphite layer (Supplementary Fig. 15). Then, the G-separators were dried at 60 °C under vacuum before cutting it into 19-mm diameter disks for cell assembly.

### Electrochemical measurements

The working electrodes were prepared by blending a mixture of active materials PTCDA (ACRROS ORGANIC), carbon nanofibers (VGCF), Denka black and polyvinyldiene fluoride (PDVF) with a ratio of 70:10:10:10 wt% in N-methyl pyrrolidone (NMP). The uniform slurry was coated onto an aluminium foil (15 µm) by a doctor blade method, dried at 120 °C under vacuum and cut into 16-mm disks. The average loading of active material was 1.5–1.7 mg cm^−2^. The electrochemical measurements were carried out using a coin-cell using a battery-grade lithium foil (16 mm diameter) counter electrode and a separator (Celgard or G-separator). Approximately 140 μl of 1M LiPF_6_ in ethylene carbonate (EC) and dimethyl carbonate (DEC) (EC/DEC: 3:7 wt.) was used as electrolyte. The cells were assembled in a glove box filled with argon. The cycled separators and lithium foil were washed with diethylene carbonate (DEC) before post mortem analysis. The electrochemical performance of the batteries was tested by Galvanostatic charge/discharge on a VMP-3 in the voltage range of 1.6–3.2 V vs Li/Li^+^ in a 25 °C oven. The electrochemical impedance spectroscopy (EIS) was carried out on a VMP-3 system at 5.0 mV ac amplitude in a 10 mHz – 200 kHz frequency range. For the measurement of the ionic conductivity, EIS was recorded for the G-separator and Celgard at 25 °C using two blocking electrodes.

### Characterization

The Fourier transform infrared (FTIR) spectra of the separators were recorded on a spectrometer Bruker Vertex 70 equipped with a smart ATR accessory. The morphology and microanalysis of the cycled anodes and separators were examined using a scanning electron microscope (SEM) operated at 5 kV coupled with a focused ion beam (FIB) with liquid Gallium (Ga) as a primary ion source (TESCAN, Lyra3 GT). Chemical analysis was performed with a windowless energy dispersive x-ray detector (Xmax 80 mm^2^ Extreme, Oxford Instruments) using 5-kV electron beam. Mapping the elemental distributions was achieved using an orthogonal time-of-flight secondary ion mass spectrometry detector (TOF-SIMS, TOF-WERK AG & TESCAN) mounted on the FIB/SEM chamber. Both positive and negative potential differences were used during the analysis to record negative and positive ions, respectively. A Gallium primary ion beam energy of 30 keV was used for the analyses with 220 pA beam current. The sample was tilted at 55° so that normal incidence with the primary ion beam and the surface of the sample was obtained.

## Supplementary information

supp info
