# Supplementary material for: Diffusion Control of Organic Cathode Materials in Lithium Metal Battery
Source: Sci Rep. 2019 Feb 4;9:1213. doi: 10.1038/s41598-019-38728-y (PMC6362235; doi:10.1038/s41598-019-38728-y)

*Supporting Information for*

**Diffusion Control of Organic Cathode Materials in Lithium Metal Battery**

Rachel L. Belanger ^1,2^, Basile Commarieu^2^, Andrea Paolella^2^, Jean-Christophe Daigle^2^, Stéphanie Bessette^2^, Ashok Vijh^2^, Jerome P. Claverie^1^ and Karim Zaghib^2,^*

^1^ Université de Sherbrooke, 2500 Blvd de l’Université, Sherbrooke, Québec J1K 2R1, Canada
^2^ Center of Excellence in Transportation Electrification and Energy Storage (CETEES), Hydro-Québec, 1800, Lionel-Boulet Blvd., Varennes, Quebec J3X 1S1, Canada

*[Zaghib.Karim@ireq.ca](mailto:Zaghib.Karim@ireq.ca)

Experimental Section

*Permeability analysis:* The permeability of the G-separator toward PTCDA was evaluated in a H-cell configuration with a PTCDA cathode, lithium metal anode and 15 ml of electrolyte solution of 1M LiPF_6_ in ethylene carbonate (EC) and dimethyl carbonate (DEC) (EC/DEC: 3:7 wt.). The G-separator and Celgard were place in between of the two compartments (anode and cathode). The graphite layer was positioned to face the cathode compartment. Aliquots were withdrawn is each of the two compartments for UV-Vis analysis using the electrolyte as the baseline spectra.

**Figures**


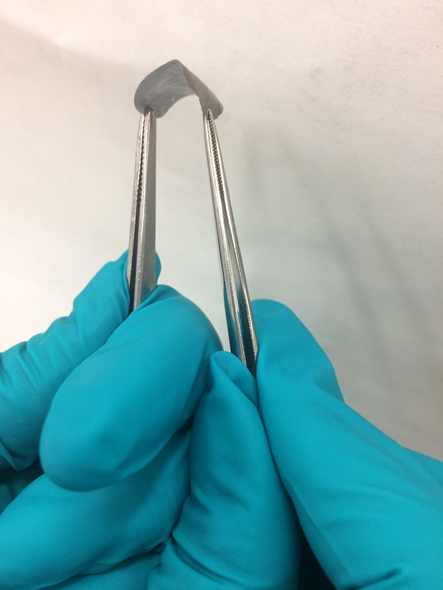


**Figure 1.** Photograph of the G-separator, which exhibits good flexibility.


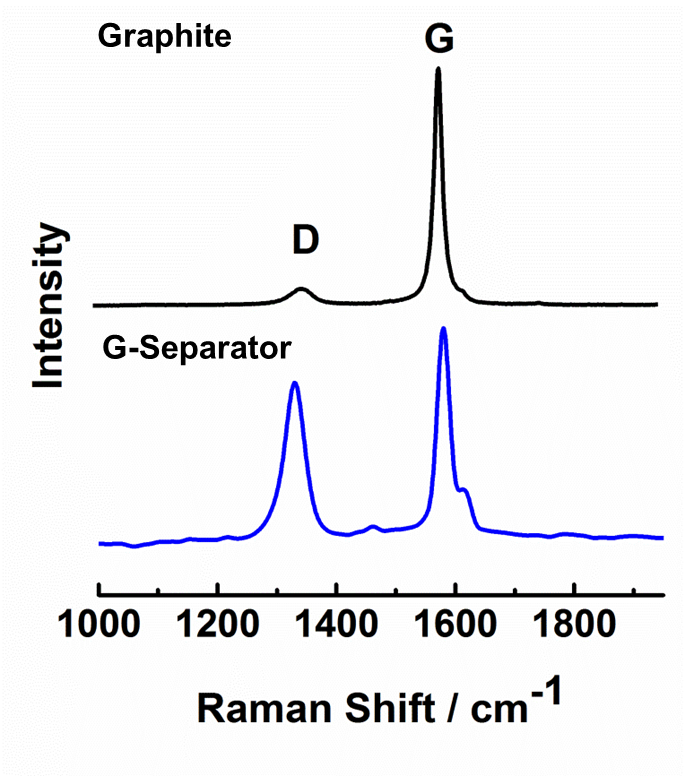


**Figure 2.** Raman spectra of the graphite powder used for the fabrication of the G-separator and the G-separator.


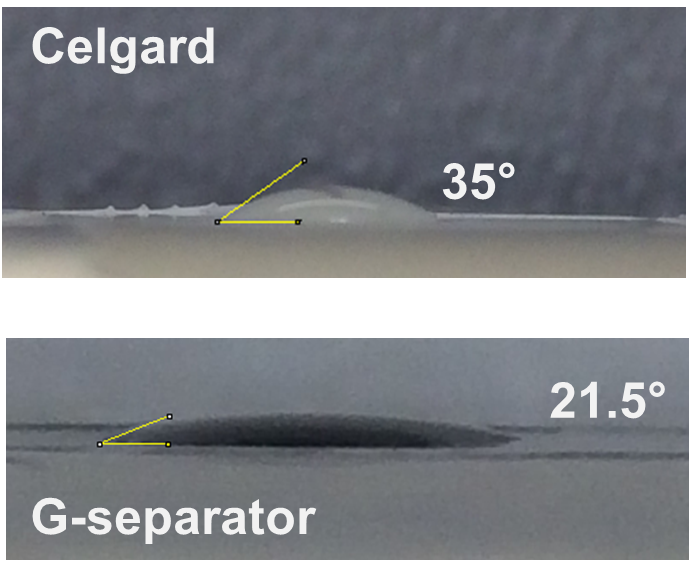


**Figure 3.** Contact angle analysis of the electrolyte (1M LiPF_6_ in EC:DEC) on the surface of Celgard and G-separator


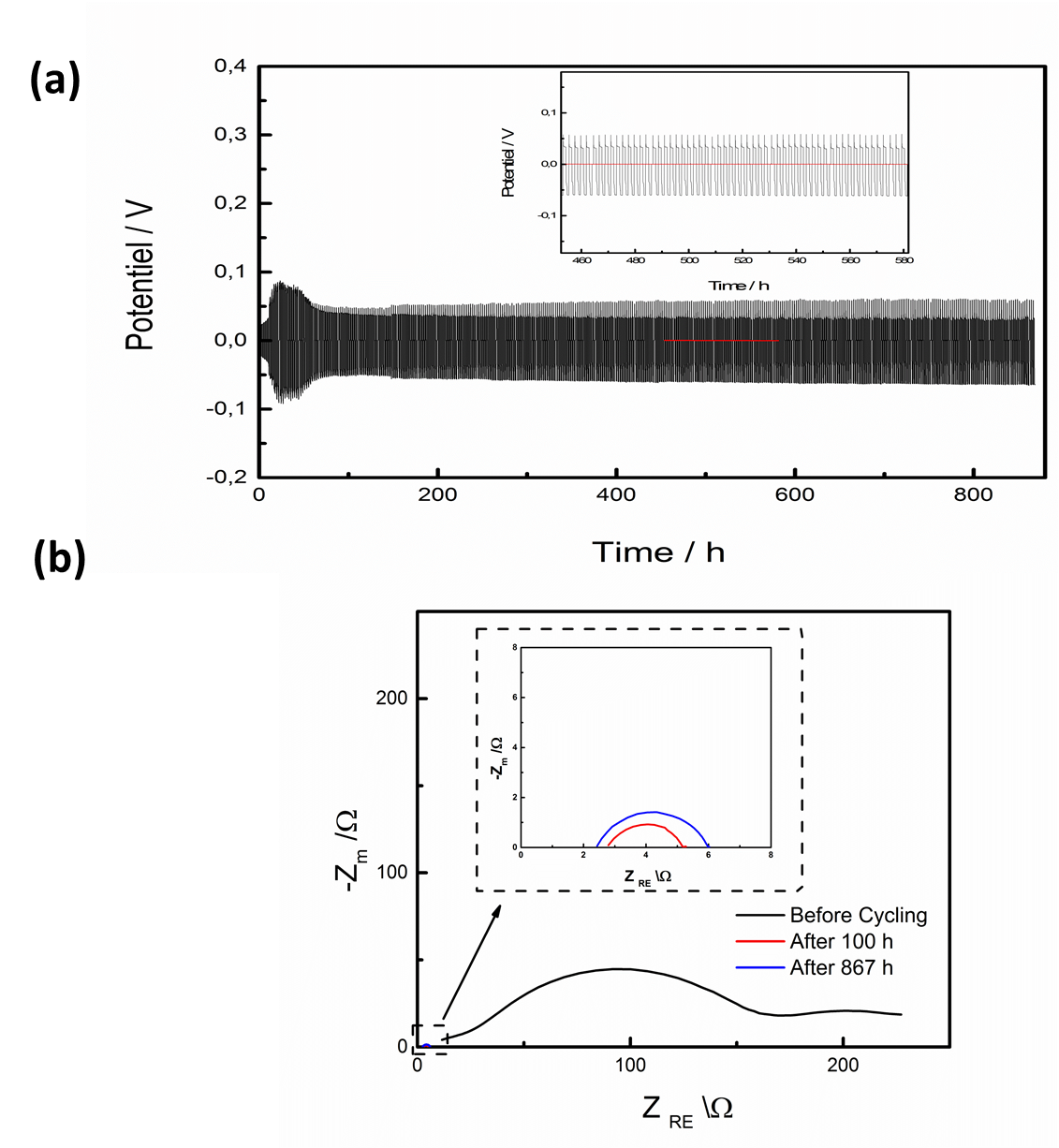


**Figure 4.** The galvanostatic cycling (a) and electrochemical impedance spectroscopy (EIS) (b) of one Li|G-Separator|Li symmetrical cell cycled at 0,5 mA cm^-2^ for 867 h. It can be seen that the dissolution and deposition curves are not perfectly symmetrical which can be cause by the two different interfaces of the cell (Li|graphite and Celgard|Li).


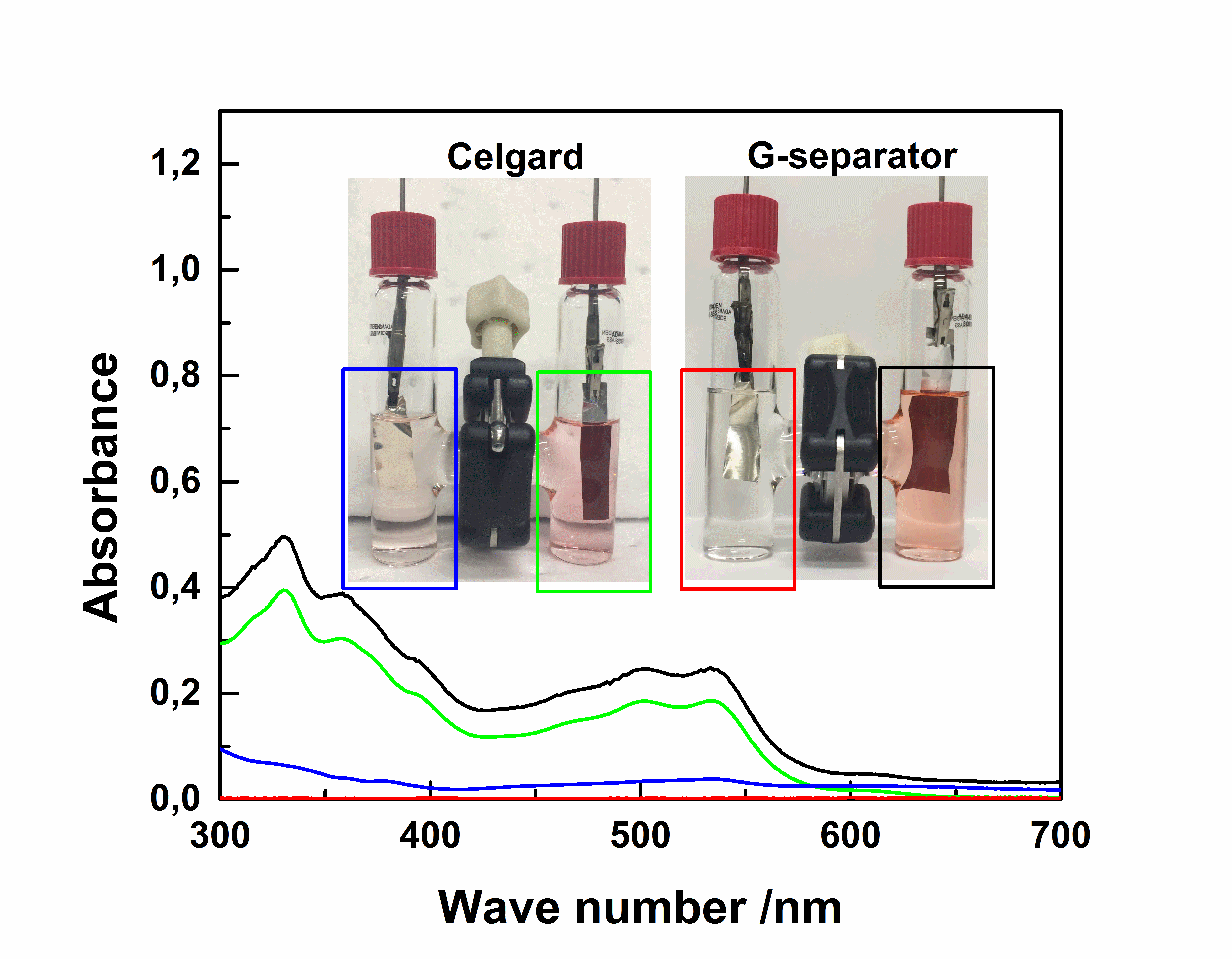


**Figure 5.** Photographs of a Li-PTCDA battery in an H-cell configuration with a Celgard separator and a G-separator, cycled for 40 hours in the potential range of 1,6 to 3,2V. Visually, PTCDA is solubilized in the electrolyte in the cathode compartment of each cell, and observed in the anode compartment of the H-cell with Celgard. The UV-Vis absorption spectra of each compartment were recorded to confirm the presence (or the absence) of PTCDA (using the color code for each curve).





**Figure 6.** The cycling performance at 1C of a Li-PTCDA battery using the G-separator A


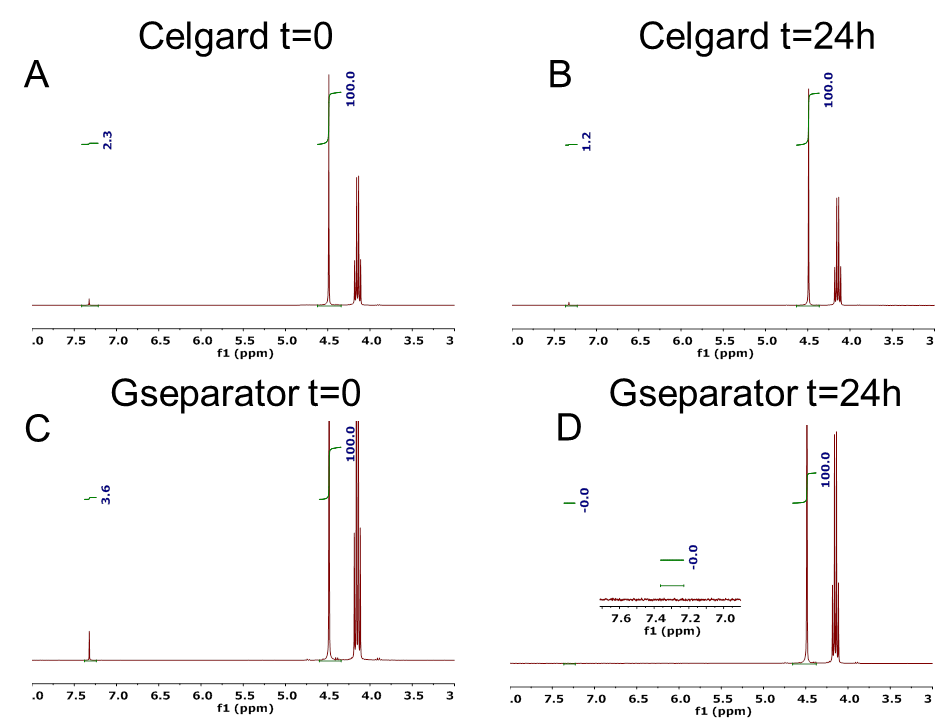


**Figure 7**. ^1^H NMR of the permeability of the G-separator to solvent molecules. An H-cell (with Celgard or G-separator) was filled with 1M LiPF_6_ in EC:DEC with no electrodes, and the cathode compartment contained 2% vinylene carbonate (VC). Aliquots were extracted initialy ( t=0) from the cathode compartment of the H-cell with a) Celgard and c) G-separator. The concentration of VC was assessed by the integral of the characteristic resonance at 7.4 ppm. After 24 h, aliquots from the anodr compartment were taken to analyse the diffusion of the VC molecules. The analysis showed no VC is found in the anode compartment of the cell using a G-separator (D, integral is 0). By contrast, an equilibrum concentration is reached using a Celgard separator (B, integral is approximatly half the integral of A).


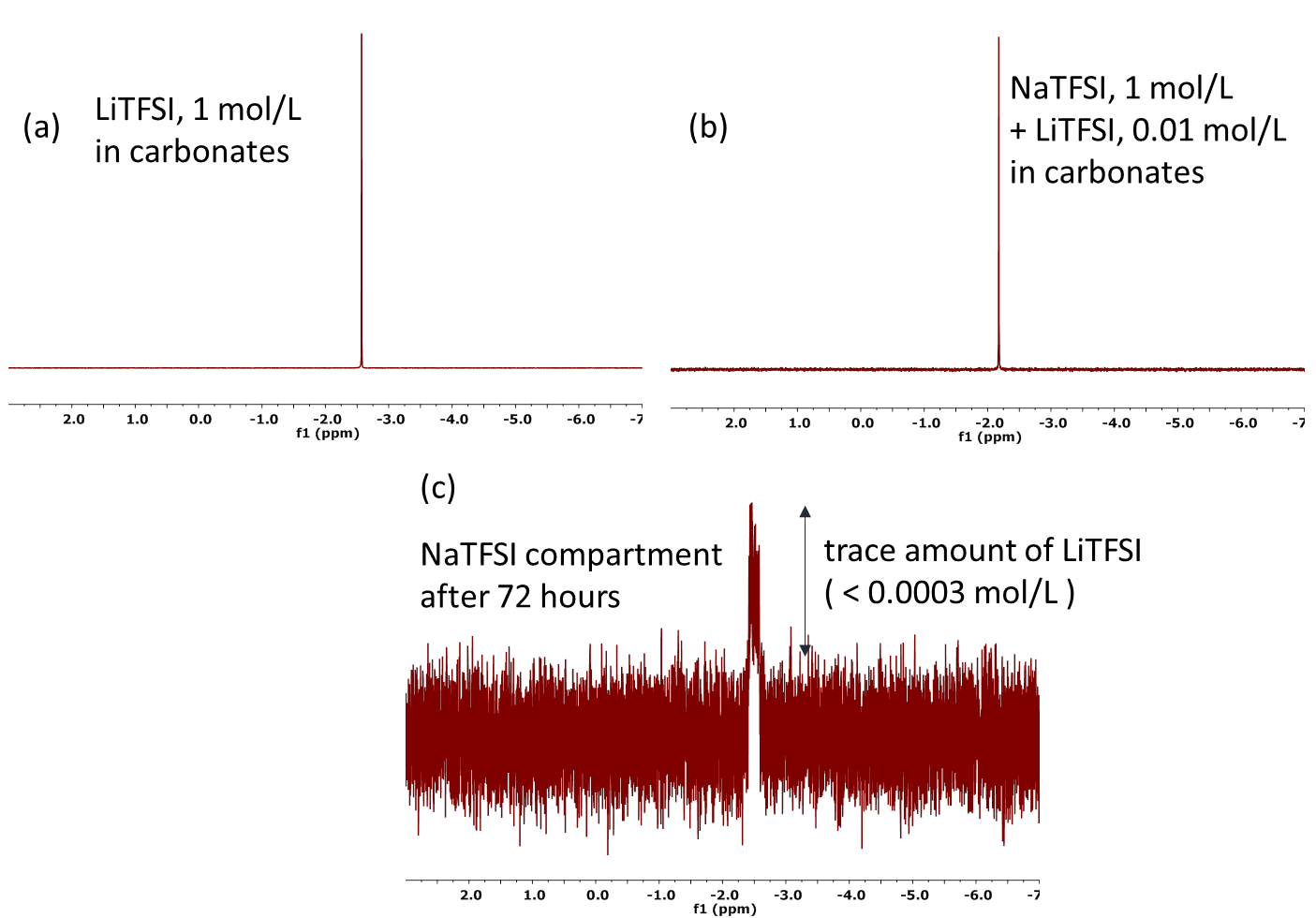


**Figure 8.** ^7^Li NMR spectra of a) compartment containing LiTFSI b) compartment containing NaTFSI with 1% of LiTFSI c) compartment containing NaTFSI after 72 hours, showing only the diffusion of a trace amount of Li^+.^  All spectra were recorded using the same conditions (8 scans, 1 Hz exponential apodization)


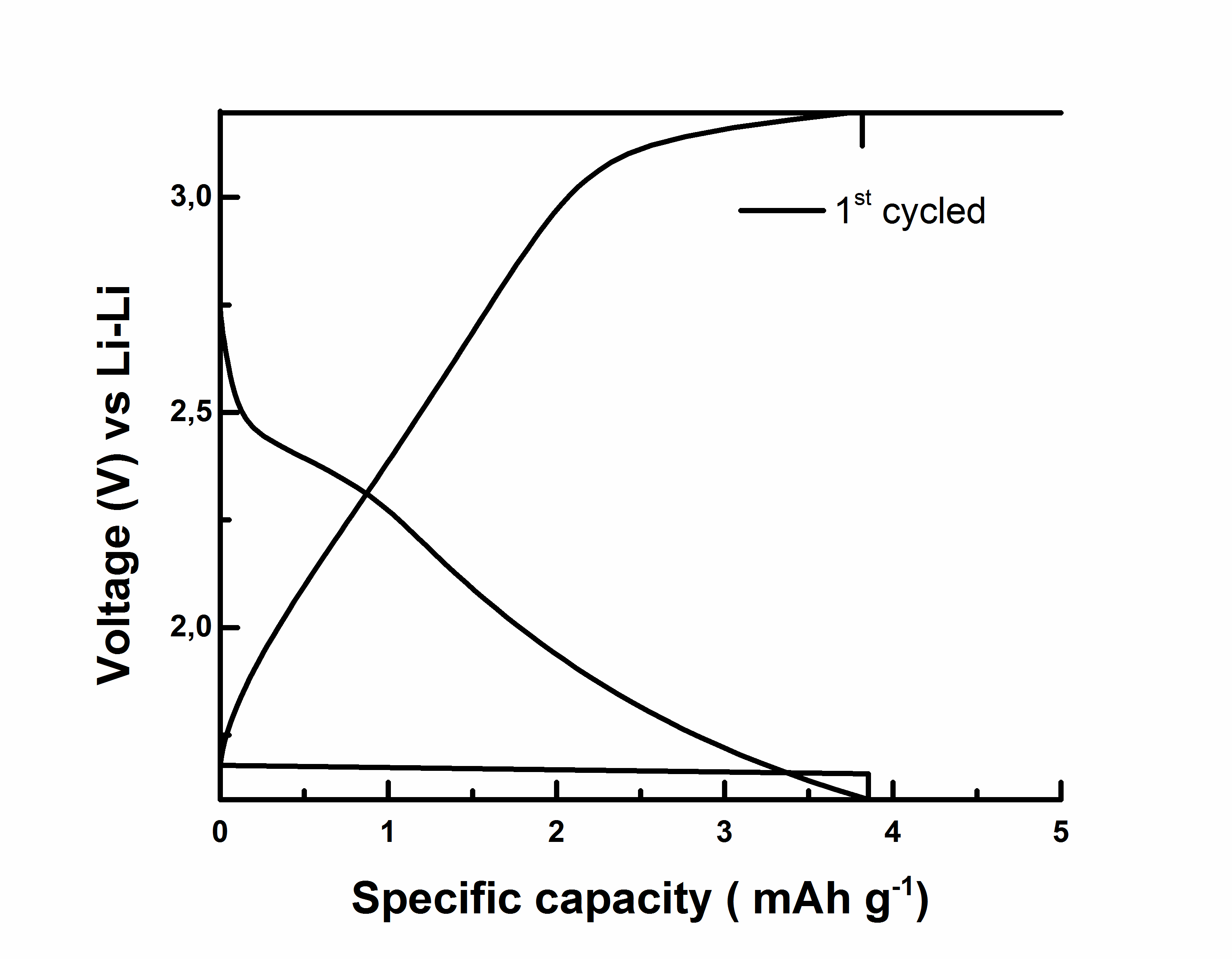


**Figure 9**. Galvanostatic cycling curve of a lithium battery using the graphite layer of the G-separator as cathode (No PTCDA cathode).


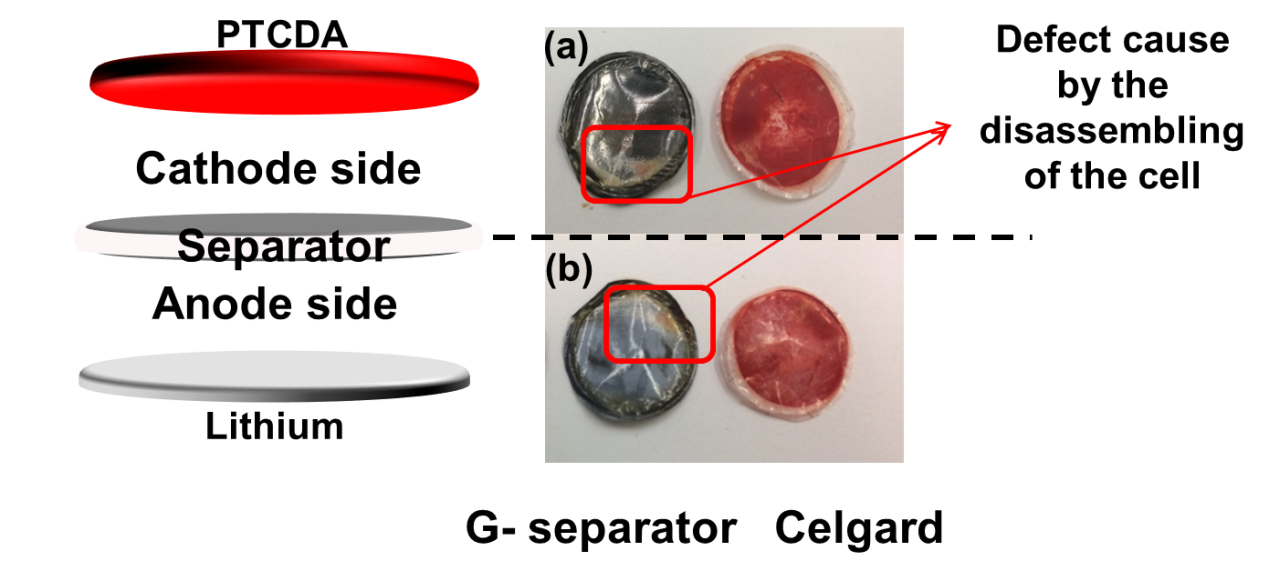


**Figure 10.** Post mortem photographs of the cycled separators (Celgard and G-separator).


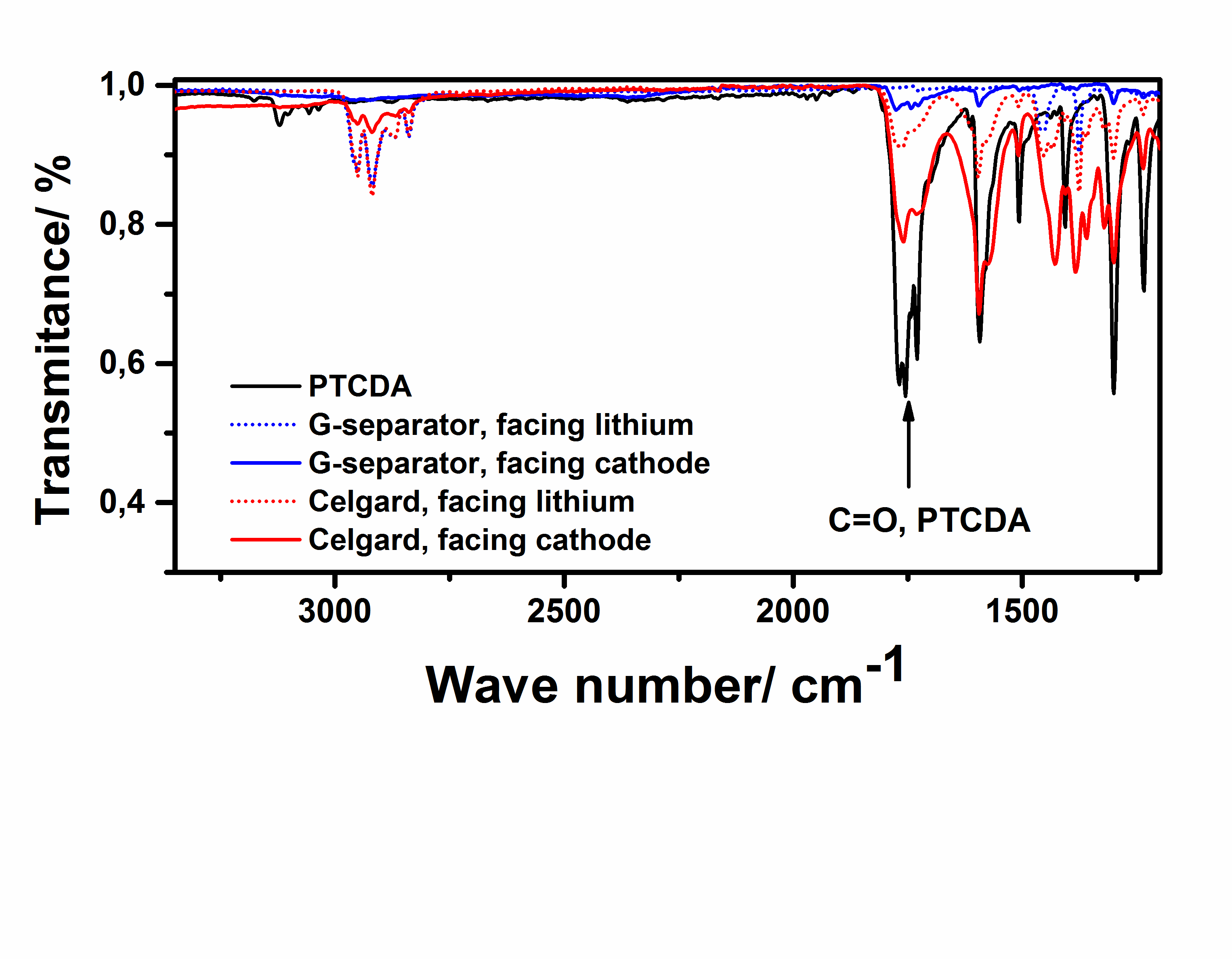


**Figure 11.** FT-IR (using attenuated total reflectance) of PTCDA powder and both sides of the cycled separator ( Celgard and G-separator)


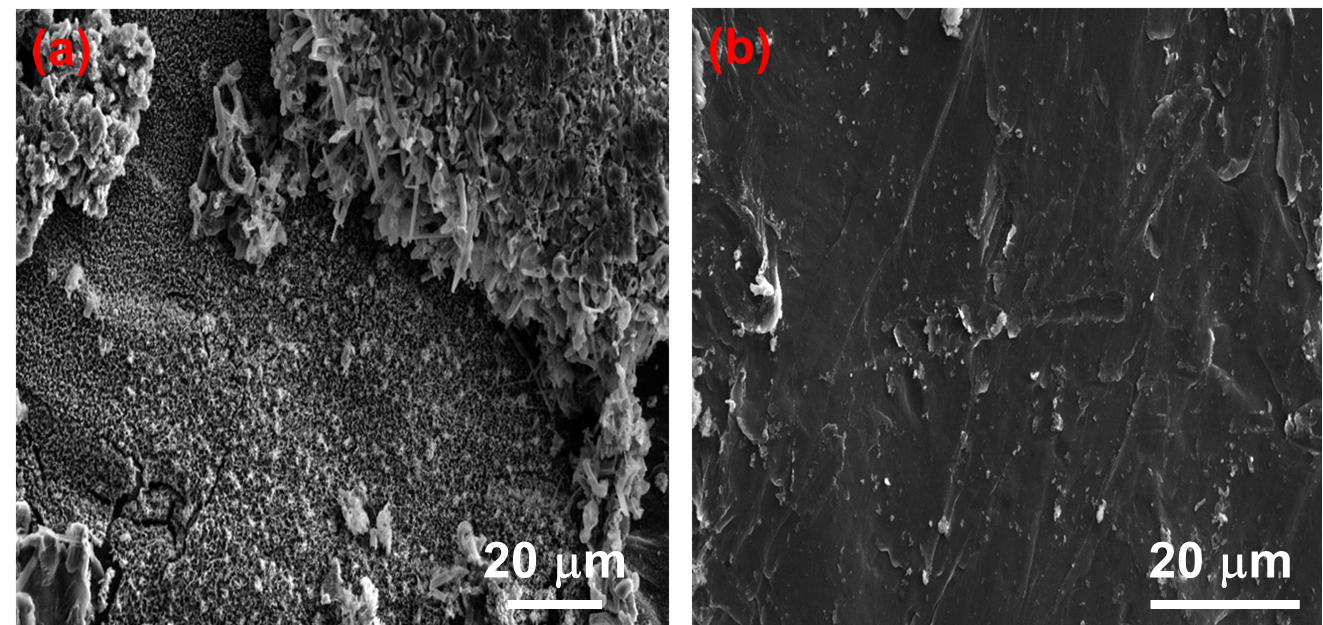


**Figure 12.** SEM images, at higher magnification, of the lithium anode cycled with the Celgard separator (a) and G-Separator (b).


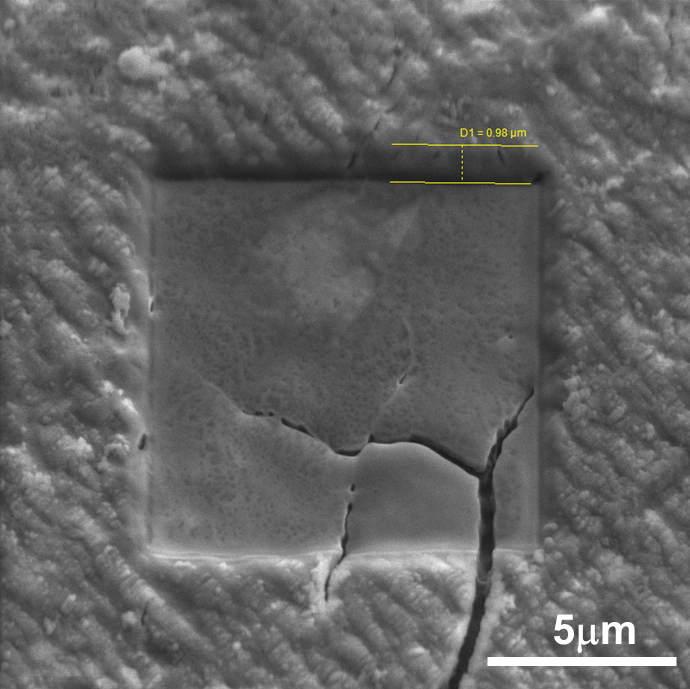


**Figure 13.** SEM images of the FIB hole resulting from the TOFF-SIMS analysis after 40 frames for the lithium anode cycled with the G-separator. An average depth of 1 μm was considered for 40 frames.


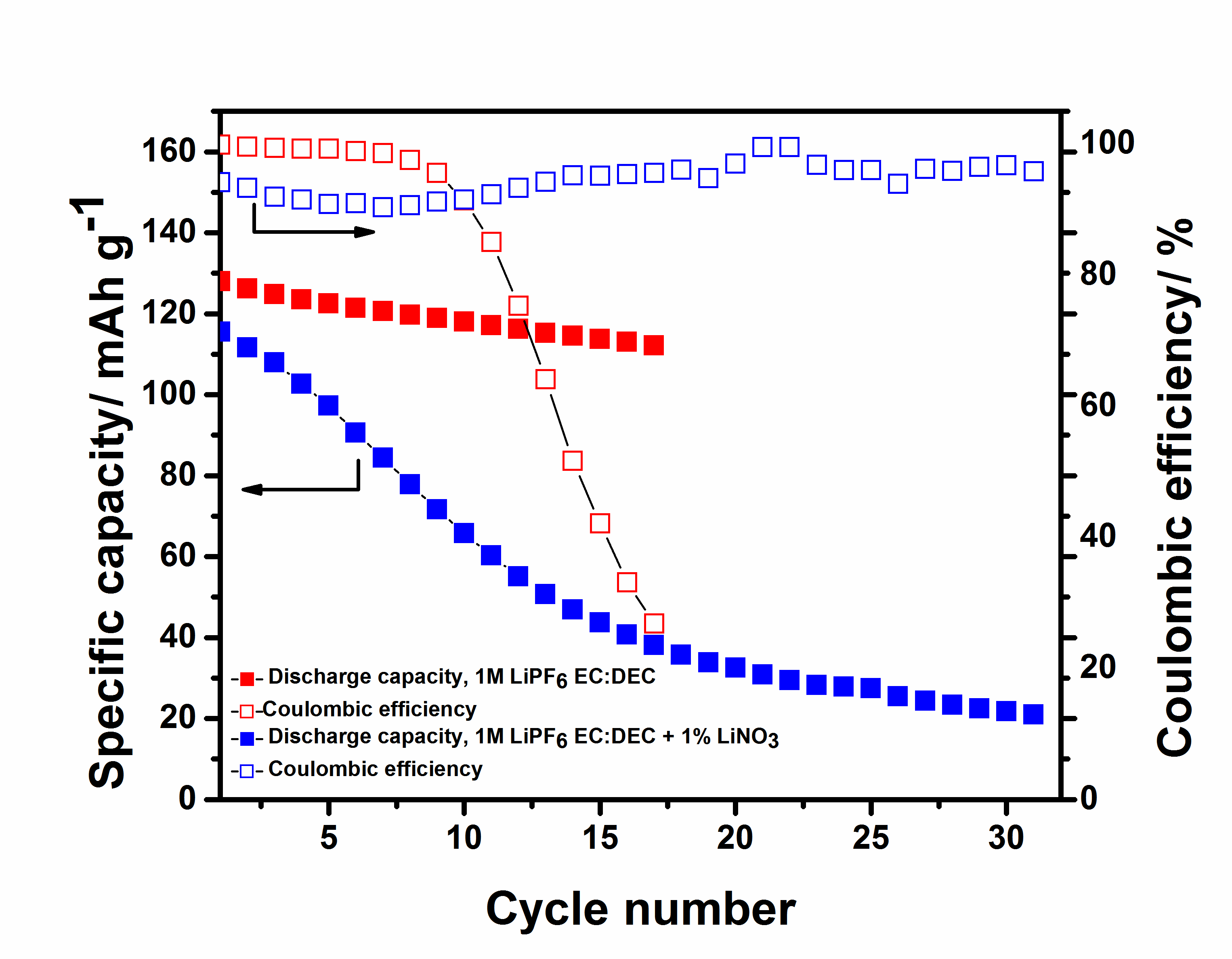


**Figure 14.** Comparison of the discharge capacity and coulombic efficiency upon cycling for two Li-PTCDA cells with 1M LiPF_6_ in EC:DEC (3:7 wt.%). One of the batteries contains 1% of lithium nitrate in the electrolyte.


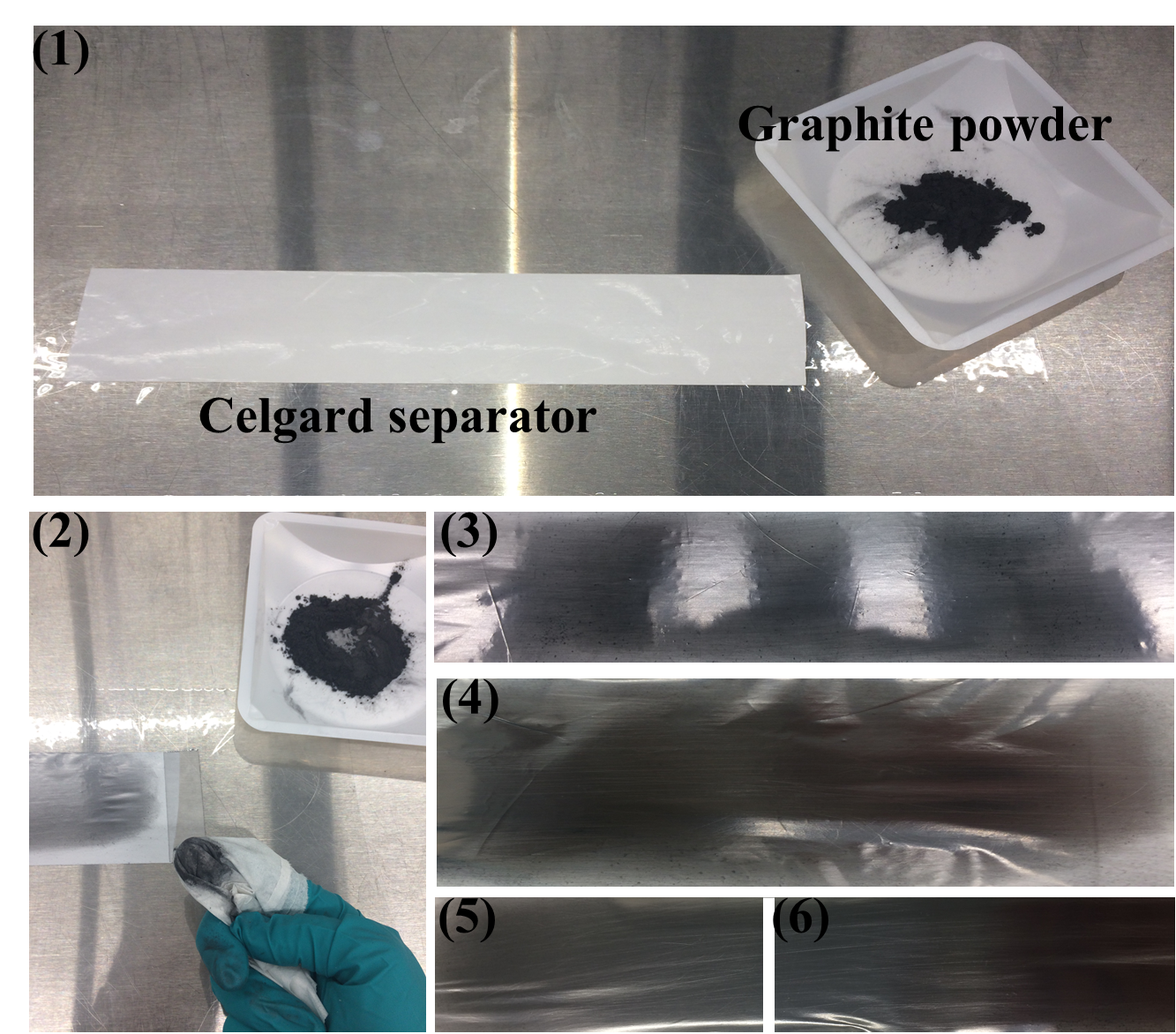


**Figure 15.** Here are the steps for the fabrication of the G-separators. The step one present the pristine celgard and the graphite used for the fabrication of the G-separators. By hand, it is possible to ‘’draw’’ a graphite layer on top of the celgard by a smearing process. It is possible to vary the thickness of the graphite layer according to the smearing time. The step three, four, five and six correspond to one, two, 5 and 10 minutes of drawing time, which can be seen by the uniformity of the graphite layer.

**Table 1:** Major fragments detected by TOF-SIMS analysis of the cycled lithium foil with Celgard separator and G-separator


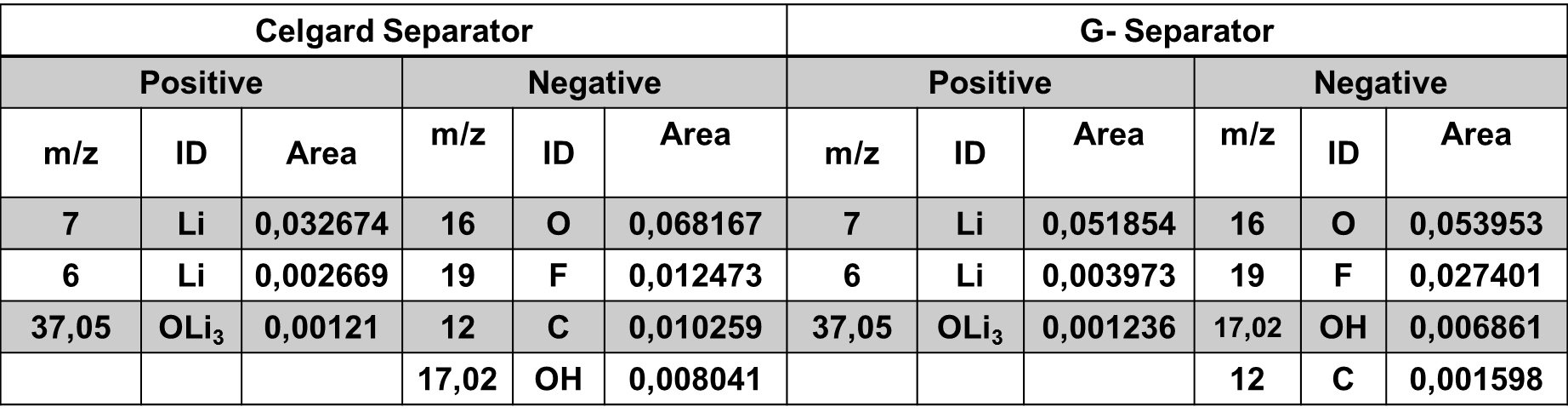

Supplement: Supplementary file 1 — supp info [file 41598_2019_38728_MOESM1_ESM.docx]
